# Peripartum Severe Acute Pancreatitis with Rare Complications: Case Report and Review of Literature

**DOI:** 10.1155/2020/5785413

**Published:** 2020-04-02

**Authors:** Syed Muhammad Ali, Nissar Ahmed Shaikh, Zia Aftab, Ejaz Latif, Muhammad Sameer, Muhammad Burhan Khan, Mohannad Al-Tarakji

**Affiliations:** ^1^Department of Acute Care Surgery, Hamad Medical Corporation, Doha, Qatar; ^2^Department of Surgical ICU Hamad Medical Corporation, Doha, Qatar

## Abstract

Peripartum pancreatitis is a rare clinical condition that occurs usually in the third trimester of pregnancy. Pancreatitis is usually secondary to gallstones, and it can lead to life-threatening and rare complications. We report a case of necrotizing postpartum pancreatitis that developed abdominal compartment syndrome (ACS) in early course, posterior reversible encephalopathy syndrome (PRES), and splanchnic and extrasplanchnic thrombosis later on. *Case*. 31-year-old female, one week after delivery, presented to the emergency department with abdominal pain, nausea and vomiting, tenderness in the epigastrium, and raised pancreatic enzymes. Ultrasound (USG) showed bulky pancreas with gallstones. She was diagnosed as having acute biliary pancreatitis and started to be hydrated and was supplemented with analgesia. Her condition deteriorated on the 2^nd^ day, and she was shifted to the surgical intensive care unit (SICU) where she developed abdominal compartment syndrome (ACS), respiratory distress, and acute kidney injury, requiring endotracheal intubation and ventilation. Computerized tomography (CT) showed pancreatic necrosis with multiple fluid collections and significant left-sided pleural effusion. Percutaneous drainage of pleural effusion was done, and she was stabilized to be weaned off from mechanical ventilation. On day 15, she underwent USG-guided drainage of the pancreatic collection and ERCP (endoscopic retrograde cholangiopancreatography) on day 19. Post-ERCP, she had tonic colonic convulsions which were treated with benzodiazepines and phenytoin. It was diagnosed by imaging studies as posterior reversible encephalopathy syndrome (PRES). Her abdomen was still distended and tender; CT showed a significant pseudocyst with splanchnic and extrasplanchnic thrombosis. She had laparotomy, gastrocystostomy, and cholecystectomy on day 28th. She made uncomplicated recovery and discharged in good health. *Conclusion*. Peripartum pancreatitis can be complicated by ACS, PRES, and splanchnic and extrasplanchnic thrombosis.

## 1. Introduction

Peripartum pancreatitis can occur during pregnancy or in the early postpartum period. It is commonly biliary in origin due to cholesterol stones and frequently seen in the third trimester and further rare in the postpartum period [1]. It can lead to various life-threatening complications. While abdominal compartment syndrome and a handful cases of posterior reversible encephalopathy syndrome (PRES) and splanchnic and extrasplanchnic thrombosis (and IVC thrombosis) have been reported in literature as complications of acute pancreatitis, we report the first case of peripartum AP complicated by ACS, PRES, and extrasplanchnic thrombosis to our best knowledge.

## 2. Case

A 31-year-old primigravida had Cesarean section delivery one week earlier that was uneventful. She presented to the emergency department with severe epigastric pain, fever (38.5°C), tachycardia (120/minute), tachypnea (25-28/minute), and normal blood pressure of 134/82 mmHg. She had upper abdominal tenderness and a clean Cesarean scar. Her complete blood count and electrolytes were within normal range, but the pancreatic amylase was 1273 units/liter (normal 13-53) and lipase of 2436 units/liter (normal 13-60). Abdominal ultrasound showed bulky pancreas with hypoechogenic areas and gallstones. She was admitted to the surgical floor and started on analgesia and hydration with a diagnosis of postpartum acute biliary pancreatitis. The next day, her condition deteriorated; she was oliguric with tachycardia and tachypnea and developed abdominal distension. She was shifted to the surgical intensive care unit (SICU).

**Figure 1:**
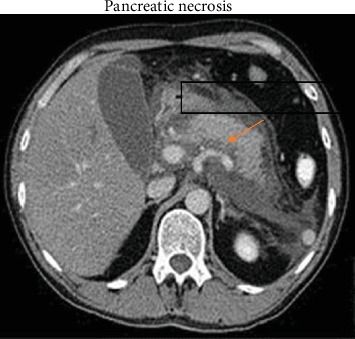
CT of the abdomen showing pancreatic necrosis (red arrow).

She was dehydrated and had elevated renal parameters. Her abdomen was distended with sluggish bowel sounds and intra-abdominal hypertension (intra-abdominal pressure 14-16 mm of Hg). She was started on invasive monitoring, and her hydration was guided by PiCCO (Pulse-induced continuous cardiac output) values and was put on noninvasive ventilation. Her chest X-ray showed left-sided pleural effusion, and computerized tomography (CT) of the abdomen revealed pancreatic necrosis with multiple fluid levels; the largest one was 5 × 6 cm (Figure 1). Her clinical condition deteriorated as abdominal pressure peaked to 25 mm of Hg, and respiratory distress worsened requiring endotracheal intubation and ventilation, and urine output decreased to <500 ml. Left-side chest tube was inserted and drained 2.1 liters of clear slightly yellow colored fluid in 24 hours. A drain was placed under radiological guidance in the retroperitoneal space, to decompress the rising abdominal pressure, and it drained 570 ml of dark, dirty brown colored fluid. She improved after the intubation and drainage as her abdominal pressure decreased (16-18 mm of Hg) and started to make 50-100 ml of urine (Table 1). She was started on nasojejunal feeding and thromboprophylaxis (dalteparin) on day six and continuously showed improvement. By the tenth day, her renal and respiratory parameters almost normalized, enteral feeding was built up to match the daily requirement, and she was extubated on day eleven and started on low-fat diet, remained hemodynamically stable, ambulated, and transferred to a surgical ward on the thirteenth day.

By the 15^th^ day, she developed fever of 38.5°C and the bilirubin increased to 53 (normal 0-21 *μ*mol/l); ultrasound of the abdomen showed dilatation of the common bile duct for which an endoscopic sphincterotomy by ERCP (endoscopic retrograde cholangiopancreatography) was performed. It showed passage of purulent bile, and no stone could be seen on fluoroscopy. Two days after ERCP, she had tonic-clonic convulsions that responded to benzodiazepines and phenytoin and shifted back to the SICU.

**Figure 2:**
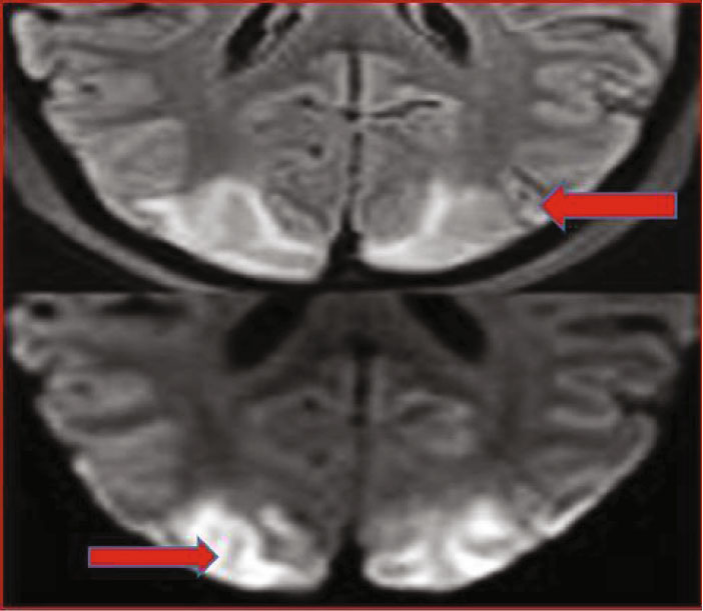
MRI of the brain showing posterior reversible encephalopathy syndrome.

She was awake, with no neurological deficit but hypertensive (180/110 mm of Hg) despite adequate analgesia and being started on lisinopril 5 mg per oral once daily. MRI (magnetic resonance image) confirmed the diagnosis of posterior reversible encephalopathy syndrome (PRES) (Figure 2). Her blood pressure was controlled, and she did not have further neurological events was transferred to the ward on the 23^rd^ day. She remained on anticonvulsant, antihypertensive, and thromboprophylaxis therapy.

**Figure 3:**
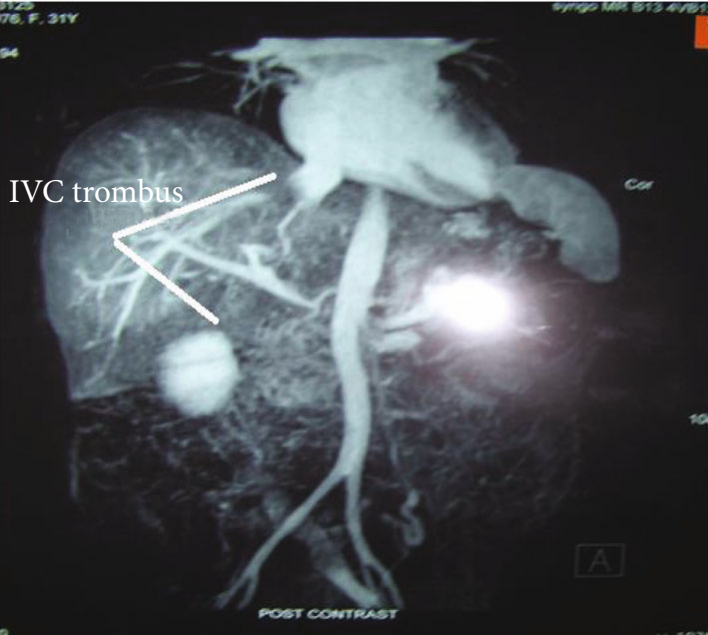
CT of the abdomen showing inferior vena cava defect.

On 27^th^ day, she had increasing abdominal discomfort, where a repeat CT of the abdomen revealed a pancreatic pseudocyst of 16 × 6 cm and necrosis with thrombosis of the splenic, portal, and superior mesenteric veins as well as the inferior vena cava (Figure 3). She underwent laparotomy, gastrocystostomy, and cholecystectomy and was nursed in the SICU. She remained normal vitally, was extubated after two days, and was started on oral diet. She was transferred to the ward on the 30^th^ day where she showed continuous improvement and was discharged home in good clinical condition, and follow-up at 6 months and 1 year remained functionally independent, on injectable insulin, oral anticoagulants (for six months), and multivitamins; however, anticonvulsants were discontinued.

## 3. Discussion

Peripartum pancreatitis occurs during pregnancy or up to 6 weeks after delivery [2]. The incidence varies from 1 in 3799 to 11467 patients [3], and biliary pancreatitis is the commonest among the causes. In older days, it was common in primigravida in the third decade of life but more recently, it is frequently seen in multiparas [4].

The etiology of peripartum pancreatitis is divided mainly in these mechanisms: (a) mechanical reflux of gastrointestinal content and obstruction due to cholelithiasis, as a result of increased serum cholesterol and formation of gallstones; (b) increased progesterone levels causing increased pancreatic secretions; (c) hypotonia of bile duct musculature and increased sphincter of Oddi tone and inflammatory and immunological interaction between the mother and fetus; and (e) adverse effects of medications particularly diuretics [2].

Therapeutic approach of peripartum pancreatitis is conservative and supportive, but laparoscopic cholecystectomy is safer despite recent delivery/Cesarean section [5]. One of the studies documents a maternal mortality of 5 to 15% whereas the recent studies do not support the notion about a perinatal mortality of 3.6% [2, 6].

Abdominal compartment syndrome (ACS) is an emergency condition defined as increased intra-abdominal pressure more than 25 mm of Hg with 2 recent organ dysfunctions. Chen et al. concluded in their study that up to 27% of acute pancreatitis patients will have ACS in the first week of hospital admission and these patients will have a higher morbidity and mortality if no decompression is done [7]. Our patient has percutaneous early decompression of fluid collection, and there is no previous report of ACS in peripartum or postpartum pancreatitis patients.

Posterior reversible encephalopathy syndrome (PRES) is a rare clinic-radiological condition commonly associated with hypertension, eclampsia, sepsis, and immunosuppressive therapy. Frequently manifested by confusional state, convulsions, and acute blindness. It is very rare in patients with acute pancreatitis, and only 8 cases of PRES are reported in the literature [8]. The systemic inflammatory response syndrome causing impaired blood brain barrier, deranged autoregulation, and vasogenic brain edema is proposed to be the etiology for PRES in acute pancreatitis patients [9], and our patient was unique to have PRES in peripartum pancreatitis.

Acute pancreatitis is an uncommon cause for portomesenteric thrombosis. In these patients, the splenic vein thrombosis occurs first and it can progress to portal vein and less commonly to superior mesenteric vein. Usually, all this thrombosis is an incidental finding during the imaging studies and it has benign asymptomatic course [10]. Extrasplanchnic venous thrombosis is a very rare finding in patients with acute pancreatitis, and it may occur in the pulmonary, the inferior vena cava, and the renal veins. Only 5 cases of inferior vena cava thrombosis are reported in medical literature [11]. Our case was very rare as the first case of inferior vena cava thrombosis in peripartum pancreatitis and 6^th^ case in acute pancreatitis to be reported. Pathophysiology for thrombosis is multifactorial and has a secondary involvement of surrounding structures by edema, inflammation, compression by pseudocyst, and intimal vascular injury due to pancreatic enzymes. Treatment of pancreatitis is conservative, and cholecystectomy is advised in the index admission.

## 4. Conclusion

Peripartum biliary pancreatitis usually has a smooth course but it can lead to a common complication such as ACS and rarely PRES and splanchnic and extrasplanchnic thrombosis.

## Figures and Tables

**Table 1:** Fluid balance and intra-abdominal pressure.

ICU day	IAP (mmHg)	Urine output (ml/hour)	Chest drain (ml/hour)	Abdominal drain (ml/24 hours)	Peak pressure (cm H_2_O)	Fluid balance (ml)
1	14-16	60	—	—	—	
2	22-25	10-20	2000	570	32-36	1520
3	16-18	50-100	210	355	24	3100
4	16	50-70	170	215	22	1200
5	16	50-70	190	200	24	1745

## References

[1] Gurjar M., Sharma A., Azim A., Baronia A. K. (2013). Acute pancreatitis in early postpartum period: a case report. *Journal of Obstetric Anaesthesia and Critical Care*.

[2] Pai P. R., Shah H. K., Samsi A. B. (1993). Post-partum pancreatitis. *Journal of Postgraduate Medicine*.

[3] Eddy J. J., Gideonsen M. D., Song J. Y., Grobman W. A., OʼHalloran P. (2008). Pancreatitis in pregnancy. *Obstetrics & Gynecology*.

[4] Ramin K. D., Ramin S. M., Richey S. D., Cunningham F. G. (1995). Acute pancreatitis in pregnancy. *American Journal of Obstetrics and Gynecology*.

[5] Hernandez A., Petrov M. S., Brooks D. C., Banks P. A., Ashley S. W., Tavakkolizadeh A. (2007). Acute pancreatitis and pregnancy: a 10-Year single center experience. *Journal of Gastrointestinal Surgery*.

[6] Joupilla P., Mokka R., Larami T. K. I. (1974). Acute pancreatitis in pregnancy. *Surgery, Gynecology & Obstetrics*.

[7] Chen H., Li F., Sun J.-B., Ji J.-G. (2008). Abdominal compartment syndrome in patients with severe acute pancreatitis in early stage. *World Journal of Gastroenterology*.

[8] Pereira V. M., Correia L. M., Rodrigues T., Faria G. S. (2016). Acute pancreatitis and posterior reversible encephalopathy syndrome: a case report. *Acta Médica Portuguesa*.

[9] Murphy T., Al-Sharief K., Sethi V., Ranger G. (2015). Posterior reversible encephalopathy syndrome (PRES) After Acute Pancreatitis. *The Western Journal of Emergency Medicine*.

[10] Easler J., Muddana V., Furlan A. (2014). Portosplenomesenteric venous thrombosis in patients with acute pancreatitis is associated with pancreatic necrosis and usually has a benign course. *Clinical Gastroenterology and Hepatology*.

[11] Patel R., Choksi D., Chaubal A., Pipaliya N., Ingle M., Sawant P. (2016). Renal vein and inferior vena cava thrombosis: a rare Extrasplanchnic complication of acute pancreatitis. *ACG Case Reports Journal*.

